# Immortalized Muscle Cell Model to Test the Exon Skipping Efficacy for Duchenne Muscular Dystrophy

**DOI:** 10.3390/jpm7040013

**Published:** 2017-10-16

**Authors:** Quynh Nguyen, Toshifumi Yokota

**Affiliations:** 1 Department of Medical Genetics, Faculty of Medicine and Dentistry, University of Alberta, 8812-112 St., Edmonton, AB T6G 2H7, Canada; nguyenth@ualberta.ca; 2 The Friends of Garret Cumming Research and Muscular Dystrophy Canada HM Toupin Neurological Science Research Chair, 8812-112 St., Edmonton, AB T6G 2H7, Canada

**Keywords:** Duchenne/Becker muscular dystrophy (DMD/BMD), antisense oligonucleotide-mediated exon skipping therapy, hDMD mice, human telomerase reverse transcriptase (hTERT), cyclin-dependent-kinase 4 (Cdk4), phosphorodiamidate morpholino oligomers (PMOs or morpholinos), C2C12, dystrophin-glycoprotein complex (DGC), golodirsen (SRP-4053), NS-065/NCNP-01

## Abstract

Duchenne muscular dystrophy (DMD) is a lethal genetic disorder that most commonly results from mutations disrupting the reading frame of the *dystrophin* (*DMD*) gene. Among the therapeutic approaches employed, exon skipping using antisense oligonucleotides (AOs) is one of the most promising strategies. This strategy aims to restore the reading frame, thus producing a truncated, yet functioning dystrophin protein. In 2016, the Food and Drug Administration (FDA) conditionally approved the first AO-based drug, eteplirsen (Exondys 51), developed for *DMD* exon 51 skipping. An accurate and reproducible method to quantify exon skipping efficacy is essential for evaluating the therapeutic potential of different AOs sequences. However, previous in vitro screening studies have been hampered by the limited proliferative capacity and insufficient amounts of dystrophin expressed by primary muscle cell lines that have been the main system used to evaluate AOs sequences. In this paper, we illustrate the challenges associated with primary muscle cell lines and describe a novel approach that utilizes immortalized cell lines to quantitatively evaluate the exon skipping efficacy in in vitro studies.

## 1. Introduction

Duchenne muscular dystrophy (DMD) is an X-linked recessive disorder affecting 1 in 3500–5000 live male births [1]. DMD is caused by mutations in *dystrophin* (*DMD*) gene located on the short arm of X chromosome (Xp21.3-p21.2) [2,3]. *DMD* is one of the largest genes in humans with 79 exons and an approximately 14 kb transcript constituting nearly 1% of the entire X chromosome [3]. DMD is caused by a variety of mutations such as deletions, duplications, small insertions/deletions (indels), and point mutations [4]. The mutation spectrum is predominated by deletions of one or more exons leading to the production of an out of frame protein resulting in an absence or insufficient amount of dystrophin and a classical manifestation of DMD. Some in-frame or truncating mutations that produce a partly functional protein lead to a milder form known as Becker muscular dystrophy (BMD) [5,6,7].

The dystrophin protein is expressed in skeletal, cardiac, smooth muscles and the central nervous system. Dystrophin has four domains: an actin-binding N-terminal domain, a rod domain consisting of 24 spectrin-like repeat motifs, a cysteine-rich domain, and a C-terminal domain. Dystrophin is localized to the muscle fiber plasma membrane in association with dystrophin-glycoprotein complex (DGC). The DGC anchors the sarcolemma to the outermost myofilament layer of myofiber, providing membrane stabilization during muscle contraction [8,9]. The complex has also been shown to function in transduction of extracellular signals to the cell’s cytoplasm [10,11].

In the absence of dystrophin, muscle fibers experience increased mechanical stress during contraction and relaxation cycles, with the sarcolemma membrane becoming fragile and susceptible to tearing and fragmentation [12]. This manifests as progressive muscle wasting and degeneration in DMD patients [13]. Additionally, this membrane instability increases intracellular calcium concentrations, thereby inducing calcium-dependent proteases and pro-inflammatory chemokines and cytokines leading to a secondary muscle degeneration and necrosis [14,15]. DMD remains largely asymptomatic for the first two years of life although affected children may show signs of delayed standing and walking. At age 3–5, clinical symptoms begin to manifest as walking abnormalities and elevated creatine kinase levels followed by generalized muscle atrophy and weakness [16,17,18]. As the disease progresses, respiratory and cardiac muscle deterioration will eventually lead to a death [2,19,20].

## 2. Exon Skipping Therapy for DMD

**Figure 1:**
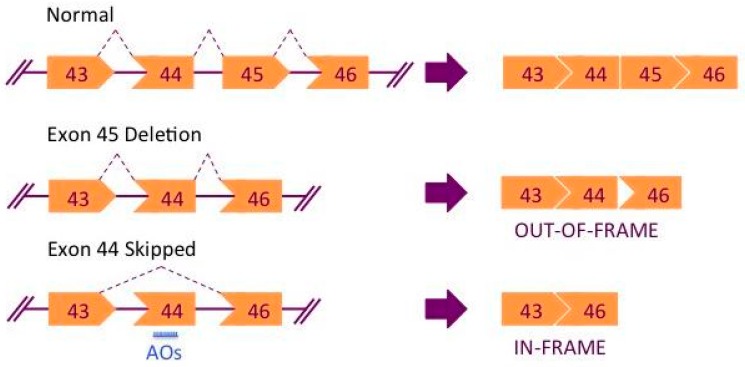
Mechanism of exon skipping therapy for Duchenne muscular dystrophy (DMD). Deletion of exon 45 results in a frameshift in the spliced mRNA. Treatment with AOs can correct the reading frame producing a truncated yet partly functional protein, as in the case of Becker muscular dystrophy (BMD).

An active body of research continues to explore therapeutic treatments to lessen the severity of DMD [21]. Currently, one of the most promising approaches is to employ antisense oligonucleotides (AOs) to induce exon skipping [22,23] (Figure 1). AOs are synthetic nucleic acid sequences that selectively bind to complementary target mRNA sequences. AOs can thereby interfere with the ribosomal complex, disrupt the splicing machinery or activate RNase H1 mediated degradation of AOs-mRNA heteroduplexes [24]. AO-mediated exon skipping can correct the reading frame by removing the mutated exon and/or its flanking exon(s) from the DMD pre-mRNA, leading to a truncated but partly functional dystrophin protein, thus producing a milder phenotype as in the case of BMD patients [25]. In animal and cell models of DMD, exon skipping has been demonstrated to correct deletion, duplication, nonsense, and splice site mutations [26,27,28,29]. An antisense phosphorodiamidate morpholino oligomer (PMO) targeting exon 51, called eteplirsen or Exondys 51 (Sarepta Therapeutics, Cambridge, MA, USA), was conditionally approved by the Food and Drug Administration (FDA) in 2016, and several PMOs targeting other *DMD* exons, including golodirsen (SRP-4053) and NS-065/NCNP-01 (NS Pharma, Paramus, NJ, USA) are currently under clinical trials. The exon skipping efficacy of different AO oligonucleotide sequences needs evaluation in vitro and in vivo. Animal models such as mice and dogs have been developed for DMD, however, each presents with its own limitations [30,31]. Naturally arising mutations in animal models that mimic human diseases are rare, and while transgenic mouse models expressing a human copy of the gene of interest have been developed, this gene expression profile remains within the context of the animal host [32,33,34]. As an example, the *mdx* mouse model for DMD exhibits a milder phenotype: their lifespan is only reduced by ~25% in contrast to humans where the disease is invariably fatal with ~75% reduction in lifespan [31]. In addition, current animal models cannot recapitulate the wide range of mutations as found in human DMD patients. For the above reasons, primary human cell cultures derived from patients’ muscle biopsies have been the pertinent tool to study the efficacy of potential therapeutic treatments prior to validation in in vivo systems. [35]. A cellular model is also less expensive and easier to set up and maintain as compared to an animal model.

## 3. Challenges with Primary Cell Models for In Vitro Studies

Primary DMD muscle cells are the most common cell type used in AO screening experiments [36]. Similar to other somatic cell lines, primary muscle cells have a limited proliferative capacity. After a certain number of cell divisions, the cells reach senescence for two primary reasons: the activation of p16-mediated cellular stress pathway, and the shortening of telomeres after each round of cell division triggering the p53 cascade and cellular apoptosis [37,38]. This limits the use of this model for repeated experiments in vitro. The proliferative lifespan is even shorter in cells derived from DMD patients due to the increased mitotic division that attempts to regenerate degenerating myofibers in vivo prior to biopsy isolations [39]. The timing of the muscle biopsy isolations can, therefore, introduce variations into the study. For repeated experiments, many isolations of primary cells are required which may result in problems with data reproducibility due to the heterogeneity associated with each biopsy procedure [40].

Prolonged in vitro cultivation may lead to deviations from normal biological processes that are important for cellular differentiation in a normal in vivo environment. One problem associated with primary muscle cell cultures is the decrease in myogenicity, and subsequently enrichment for non-myogenic cells with successive passaging. S. Perie et al. showed the percentage of desmin-positive cells (a marker specific for myogenic cells) decreased significantly during primary cell cultures’ proliferative lifespan [41]. Although the study was done on cells derived from oculopharyngeal muscular dystrophy patients, it is reasonable to expect a similar decline in the case of other muscular dystrophies.

Quantification of dystrophin expression level is the criterion by which the effectiveness of in vitro exon skipping is evaluated. Primary muscle cells prove to be challenging in this regard as they may not express sufficient amount of dystrophin mRNA and protein for quantification. Previous studies have tried to overcome this limitation by using an extra cycle of PCR to quantify the first PCR cycle’s products (nested PCR) [42,43,44,45]. However, this approach is likely to overestimate dystrophin levels, and the results are often not reproducible. Difficulties with accurate dystrophin quantification limit the potential to screen for more effective AO sequences for use in vitro, in vivo studies, and ultimately clinical trials. Since the cell type used for in vitro studies of dystrophin exon skipping efficacy could significantly affect the result, finding the appropriate system within which to test AOs has proved an active area of research.

Previous studies have constructed cell models in which a plasmid harboring the native human dystrophin sequences was introduced [46]. This artificial screening cell model is easier to maintain than primary cells due to their higher proliferative capacity. However, due to the large size of human DMD gene’s introns, it was not possible to incorporate the entire gene into a plasmid. Only selected exons were introduced into a plasmid, and some introns were replaced in these models, therefore, the efficacy of exon skipping might not reflect the in vivo efficacy. Additionally, protein expression cannot be measured in these models. Alternatively, to overcome insufficient mRNA and protein expression in primary cell lines, MyoD-transduced fibroblasts have been employed in exon skipping assays [47]. Dystrophin could be detected seven days after differentiation in dog cells, but required two weeks or more in human cells. MyoD-transduced fibroblasts also showed significantly higher dystrophin expression, and the results were reproducible. While MyoD-transduced fibroblasts were demonstrated to be a suitable model to study exon skipping efficacy, it required transduction by virus vectors, which is labor-intensive and time-consuming.

## 4. Immortalized Cell Lines as a New Tool for In Vitro Studies

To overcome problems with quantification, our group’s recent studies have demonstrated the feasibility of quantifying dystrophin in immortalized DMD cell lines [33,48]. Muscle cells derived from DMD patients were transduced with human telomerase reverse transcriptase (hTERT) and cyclin-dependent-kinase 4 (CDK4)-expressing vectors to generate muscle stem cell lines with an enhanced proliferative capacity. hTERT is capable of elongating telomeres after each round of cell division while CDK-4 blocks the activation of p16-mediated cellular stress pathway that together lead to cell cycle senescence [40,49]. These immortalized cell lines have been previously tested to confirm that they maintained their myogenic signature and retained the ability to differentiate into myotubes [40]. Immortalization has also been shown to have no effect on other cellular processes [50].

In an effort to develop an in silico screening tool to predict the optimal target sites for exon skipping, our group utilized immortalized cell lines for subsequent in vitro testing to validate the predictive algorithm [48]. These cell lines had a high proliferative capacity and eight days after differentiation, sufficient mRNA and protein could be harvested to quantify expression levels of both by RT-PCR (non-nested) and Western blotting. To avoid overestimating the level of dystrophin, expression levels in treated DMD cells were calculated using a standard curve prepared from 1 to 10 percent protein of immortalized healthy skeletal muscle cells at nine days after differentiation as a positive control. The study found that novel AOs targeting exon 53 and exon 44 restored dystrophin expression to 5 and 12 percent of normal dystrophin expression levels in DMD muscle cells in vitro. We also observed a strong correlation of in vitro level of exon skipping and the predicted exon skipping efficacy calculated by our in silico screening tool, with an average *R*^2^ of 0.89 for both exon 53 and 44. Observed dystrophin protein level also correlated well with predicted values, at an average *R*^2^ of 0.78 for exon 53 and 0.73 for exon 44. Of the parameters tested, the binding energetics of the oligonucleotide to the RNA and the distance in bases of the target site from the splice acceptor site were confirmed to be the two most predictive.

In 2016, the FDA approved the first AO based drug, eteplirsen, designed for DMD exon 51 skipping. The FDA’s approval of the drug remains a controversial decision; there is a dearth of evidence to support the effectiveness of eteplirsen in terms of its therapeutic effects in patients [51,52,53,54]. The FDA had previously rejected another candidate drug for DMD, 2’-*O*-methyl-phosphorothioate-based AO drisapersen, due to concerns over its therapeutic effectiveness and safety [55]. Previously, AO screenings relied heavily on RT-PCR from primary DMD muscle cell to evaluate dystrophin in-frame mRNA rescue level. As both drisapersen’s and eteplirsen’s exon 51 skipping efficacy was evaluated using this method, a more accurate methodology can, therefore, be used to identify even better target sequences [36,42]. Using our in silico screening tool to design AOs targeting DMD exon 51, and employing immortalized DMD muscle cell lines to quantitatively evaluate the exon skipping efficacy in vitro, we were able to find two new AOs sequences that induced exon skipping and rescued dystrophin expression with 12- and 7-fold greater efficiency than eteplirsen [33,48,56]. In this study, immortalized cell lines were generated from muscle cells of three healthy subjects and two DMD patients bearing deletions of exon 52 and exons 48–50. All healthy cell lines produced detectable levels of dystrophin three days after differentiation. A positive control from a healthy subject with the highest level of dystrophin was used to avoid overestimation of dystrophin expression. RT-PCR results using these cell lines found five novel sequences that showed significantly higher exon skipping efficacy compared to sequences identical to either eteplirsen (analog eteplirsen) or drisapersen (analog dirsapersen). In particular, treatment with a 30-mer AO targeting the beginning of exon 51 (called Ac0) had the highest proportion of in-frame transcripts at 72% of total dystrophin transcripts, 4 and 25 times higher than analog eteplirsen and drisapersen, respectively. Ac0-treated cells also had the highest level of dystrophin expression at 16% of wild-type followed by Ac48-treated cells at 13% as quantitated by Western blotting. Additionally, treatment with Ac0 or Ac48 showed more dystrophin positive myotubes than either analog eteplirsen or drisapersen in immunocytochemistry assays. The results were confirmed in primary muscle cell lines and in vivo in transgenic mice harboring the human *DMD* gene (hDMD mice). Exon skipping efficacy was analyzed by RT-PCR in tibialis anterior muscles two weeks after intramuscular injection of Ac0 or analog eteplirsen. Ac0 induced over 7% exon skipping, compared to less than 5% in analog eteplirsen injected mice. These experiments also demonstrated for the first time that the observed exon skipping efficacy correlated well with the amount of dystrophin rescued. Therefore, we demonstrate that immortalized cell lines have the potential to be a powerful screening tool for identifying new sequences yielding higher exon skipping efficacy, and one that overcomes many of the challenges presented by dystrophin quantifications.

## 5. Considerations Regarding Immortalized Cell Lines

Several groups, including our own, have shown immortalized cell lines to be an invaluable tool to study cellular and molecular mechanisms in a disease model [33,40,48,57]. Thorough evaluations are nevertheless required to confirm the lines’ stability and ability to retain the characteristics of the unmodified parental population. Since immortalization involves the introduction of exogenous DNA into the cells and overexpression of one or more cellular components, this may significantly change a culture’s characteristics. The previously described hTERT, for example, has been reported to possess putative non-telomeric activities; evidence points to its interference with the Wnt pathway in mice essential for cellular differentiation [58,59,60,61,62]. The second target, CDK4, possesses key roles in the regulation of the cell cycle and other fundamental cellular processes. It is therefore essential to determine if genetic modifications related to CDK4 have secondary effects that could affect the validity of these models. Another consideration is that immortalized cell lines, unlike primary cell lines, are likely to be maintained for prolonged periods in tissue culture conditions. Time in tissue culture has been associated with loss of myogenic potential, thus further emphasizing the necessity of validating immortalized cell lines’ divergence from their primary parental population [41]. Phenotype drifts have been observed in other immortalized cell lines such as in the case of C2C12 cells, a phenomenon that could result in data reproducibility problems [40].

Although in vitro models remain an approximation of the level of complexity of a disease in a human body, immortalized cell lines prove to be a powerful tool for therapeutic preclinical studies.
